# Completion Time Minimization for Multi-UAV Information Collection via Trajectory Planning

**DOI:** 10.3390/s19184032

**Published:** 2019-09-18

**Authors:** Zhen Qin, Aijing Li, Chao Dong, Haipeng Dai, Zhengqin Xu

**Affiliations:** 1 College of Communications Engineering, Army Engineering University of PLA, Nanjing 210042, China; qzqzla912@163.com (Z.Q.); lishan_wh@163.com (A.L.); zhengqinxu66@163.com (Z.X.); 2 College of Electronic and Information Engineering, Nanjing University of Aeronautics and Astronautics, Nanjing 211106, China; 3 Department of Computer Science and Technology, Nanjing University, Nanjing 210023, China

**Keywords:** wireless sensor networks, unmanned aerial vehicle, mission completion time, trajectory planning

## Abstract

Unmanned Aerial Vehicles (UAVs) are widely used as mobile information collectors for sensors to prolong the network time in Wireless Sensor Networks (WSNs) due to their flexible deployment, high mobility, and low cost. This paper focuses on the scenario where rotary-wing UAVs complete information collection mission cooperatively. For the first time, we study the problem of minimizing the mission completion time for a multi-UAV system in a monitoring scenario when considering the information collection quality. The mission completion time includes flying time and hovering time. By optimizing the trajectories of all UAVs, we minimize the mission completion time while ensuring that the information of each sensor is collected. This problem can be formulated as a mixed-integer non-convex one which has been proved to be NP-hard. To solve the formulated problem, we first propose a hovering point selection algorithm to select appropriate hovering points where the UAVs can sequentially collect the information from multiple sensors. We model this problem as a BS coverage problem with the information collection quality in consideration. Then, we use a min-max cycle cover algorithm to assign these hovering points and get the trajectory of each UAV. Finally, with the obtained UAVs trajectories, we further consider the UAVs can also collect information when flying and optimize the time allocations. The performance of our algorithm is verified by simulations, which show that the mission completion time is minimum compared with state-of-the-art algorithms.

## 1. Introduction

**Figure 1:**
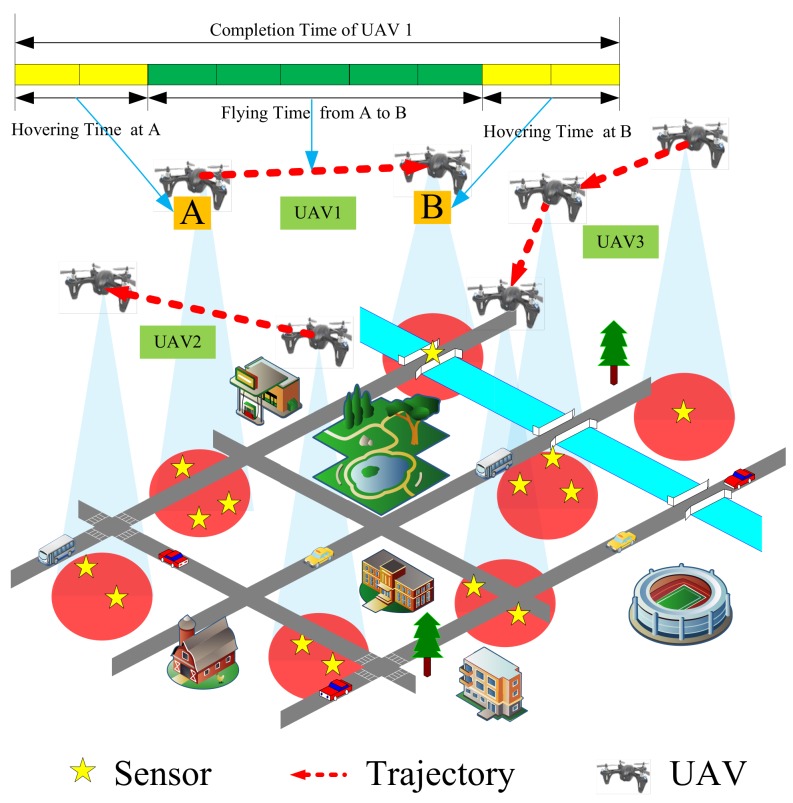
Monitoring scenario.

Due to their tremendous application potentials in military and civilian related applications, Wireless Sensor Networks (WSNs) have attracted increasing research interest in many fields, such as industrial process control, environmental monitoring, and battlefield surveillance  [1,2,3,4]. Generally, WSNs are composed of small, battery-limited sensors which cannot transmit over a long distance  [5]. There is a great interest recently in utilizing rotary-wing Unmanned Aerial Vehicles (UAVs) as mobile information collectors in WSNs  [6,7,8]. They can move towards the sensors and establish reliable connections with them due to their high mobility and flexible deployment  [9]. Rotary-wing UAVs are equipped with propellers that enable them to hover over fixed locations. This advantage makes them suitable candidates for mobile information collectors as they can hover over sensors to collect information  [10,11]. Nowadays, with the popularity of WSNs, a single UAV cannot meet the demands of monitoring missions over larger monitoring areas. Information collection of WSNs with multi-UAV cooperation is more desired. Currently, there are two types of methods to collect the information from WSNs with multiple UAVs. The first is to use static UAVs to form a network to cover all sensors, which is suitable for small area monitoring  [12,13,14,15]. The second is to use mobile UAVs to cover a larger area  [16,17,18]. As shown in Figure 1, we mainly focus on the second scenario in this paper.

For many WSNs applications, the timeliness of monitoring information is critical. Therefore, the mission completion time of multi-UAV cooperative information collection is very important. For example, in a city security monitoring system, if the control center gets fire information as soon as possible, the city may avoid serious economic loss and casualties. In traffic monitoring applications, real-time road information helps cars effectively avoid traffic congestion. In this paper, the mission completion time includes flying time and hovering time, both of which are related to the trajectory planning of the UAVs. Therefore, the trajectories need to be carefully planned such that UAVs can complete theirs missions quickly and satisfy the communication requirements along their entire trajectory. By optimizing the trajectory of each UAV, we pursue minimizing the mission completion time of multi-UAV system.

Some works have studied how to minimize a single UAV’s the mission completion time  [16,17,19,20,21]. However, these works cannot be used for information collection with multi-UAV directly. In fact, a multi-UAV system requires solving two sub-problems, task assignment and trajectory optimization. Generally, we cannot address one of problems individually because the two sub-problems are tightly coupled. If task assignment is not considered in trajectory planning, it may result in unreasonable task assignment. For example, a UAV may be responsible for too many tasks, which can lead to longer mission completion time. If trajectory optimization is not considered in task assignment, it may result in long flying distance, which may also lead to longer mission completion time. Therefore, we cannot solve the two sub-problems separately. In other words, we cannot assign tasks first, and then apply the algorithm of a single UAV to solve this problem. Since we use a multi-UAV system to collect information, the completion time of the entire mission is the time of all tasks completed by UAVs. In other words, it is the maximum completion time among the UAVs. The completion time of a UAV is mainly divided into two parts, flying time and hovering time. As shown in Figure 1, we give a toy example illustrating flying time and hovering time. The flying time is the time taken for the UAV to fly from point A to point B. The hovering time is the time for the UAV staying at the hovering point (A or B) to collect information from sensors. There are two main differences from previous works. First, some works only consider flying time or only consider hovering time in UAV-enabled network  [22,23,24]. Second, some works assume that UAVs only collect information from sensors when hovering  [25,26,27,28,29,30]. If flying time is fully utilized, the mission completion time can be reduced. Therefore, we consider that the UAVs can collect information when they are hovering and flying.

In this paper, our objective is to plan the trajectories of all UAVs to minimize the mission completion time, while ensuring that the information of each sensor is collected. Rotary-wing UAVs can collect information when they are hovering and flying. This problem can be formulated as a mixed-integer non-convex problem. Since the problem involves infinitely many variables over time, it is difficult to optimize trajectories, and hovering and flying communication scheduling at the same time. To tackle this problem, we propose an improved fly-hover-fly trajectory planning algorithm. We first propose a hovering point selection algorithm to get hovering points. Then, based on these hovering points, we plan the trajectory of each UAV. Intuitively, it can use the algorithm of Multiple Traveling Salesman Problem (MTSP) to plan the trajectories of multi-UAV. However, MTSP’s goal is to minimize the total completion time of multi-UAV. Different from MTSP, our problem aims to collect all sensors’ information with multi-UAV such that the maximum completion time among UAVs is minimized. By using the Base Station (BS) coverage algorithm and min-max cycle cover algorithm, we select appropriate hovering points where the UAVs can sequentially collect the information from multiple sensors and get the trajectory of each UAV. Finally, based on the obtained trajectories, we propose an effective method to optimize UAVs hovering and fly-collection time allocations. The main contributions of this paper are summarized as follows:

- To the best of our knowledge, this is the first paper to study how to minimize the mission completion time considering the information collection quality when multiple rotary-wing UAVs collect information cooperatively in WSNs. We model it as a mixed-integer non-convex problem, which has been proved to be NP-hard.
- We first propose the FHF algorithm to optimize the hovering points and obtain the trajectories. We select appropriate hovering points where the UAVs can sequentially collect the information from multiple sensors. We model this problem as a BS coverage problem with the information collection quality in consideration. Then, we use a min-max cycle cover algorithm to assign these hovering points and get the trajectory of each UAV.
- With the obtained UAVs trajectories, we further consider that the UAVs can also collect information when flying. We propose an effective method to optimize UAVs hovering- and fly-collection time allocations.
- Extensive simulation results show the mission completion time of the proposed algorithm is minimum and closer to the optimal scheme compared with other algorithms. Furthermore, we also verify the performance of the algorithms under different settings.

The rest of this paper is organized as follows. In Section 2, we introduce the related work. The system model and problem formulation are presented in Section 3. Then, we propose the FHF algorithm to optimize the hovering points and obtain the trajectories in Section 4. In Section 5, we optimize the time allocations. Simulation results are provided and analyzed in Section 6. Discussion is provided in Section 7. Finally, we conclude the paper in Section 8.

## 2. Related Work

### 2.1. Single UAV’s Mission Completion Time Problem

There are many studies on minimizing a single UAV’s mission completion time. In  [16], the authors studied a UAV-enabled multicasting system and planned the trajectory of the UAV for minimizing mission completion time, while ensuring that ground terminals successfully recovered files. To reduce the single UAV’s mission completion time and periodic flight duration, Zhang et al.  [17] optimized the trajectory of UAV and communication resource allocation, while satisfying the data requirement of ground users. Zhang et al. [19] focused on a scenario where a single UAV communicated with multiple ground base stations. They aimed to minimize the mission completion time by planning the trajectory of UAV, while the UAV was also subject to practical communication connectivity constraint with ground base stations. One of the most important reconnaissance missions of the UAV is to take pictures of interesting targets in a wide area. Under this scenario, Lim et al.  [20] proposed a trajectory generation algorithm for reconnaissance UAV with time constraints. Zhang et al.  [21] proposed sparse $A^{*}$ search approach for a single UAV route planning with time constraints. It can be seen that the above works only discuss a single UAV’s mission completion time in different scenarios. However, these works cannot be used for information collection with multi-UAV directly. Therefore, the mission completion time problem of multi-UAV is worth being further studied.

### 2.2. Multi-UAV’s Mission Completion Time Problem

There are some studies on completing missions by multi-UAV within the specified time. Pohl et al.  [31] studied the routing of multi-UAV, while the trajectories of UAVs met the constraints of total path cost optimization, total mission time, enemy radar avoidance, and time on target. This work aimed to minimize the total distance by planning trajectories. Shi et al. [32] proposed a statistical physics method for UAVs cooperative reconnaissance mission planning, while the trajectories satisfied the reconnaissance time requirements. However, in many applications, it is more important to complete the missions as soon as possible. In  [23], the authors introduced a geometric path planning method and proposed a resource allocation algorithm for multi-UAV. However, they only considered the flying time. Hayat et al. [33] optimized the trajectory of each UAV and synchronized multi-UAV so that the UAVs can fly to the next task only after all UAVs had completed their previous task. Mousavi et al.  [34] aimed to form UAV coalition formation to reduce the mission completion time. Wang et al. [35] found the optimal schedule to perform diverse missions of different time windows at various locations using fixed-wing heterogeneous UAVs. The solutions of minimizing the mission completion time for multi-UAV system are not directly applicable to our problem.

## 3. System Model and Problem Formulation

### 3.1. System Model

As shown in Figure 1, we consider a monitoring scenario where multiple rotary-wing UAVs are employed to collect information from sensors whose distributions follow the homogeneous Poisson Point Process. Traditionally, sensors transmit their monitored information to a base station via multi-hop transmissions. Therefore, a sensor is required to not only transmit its own information, but also relay the information of other sensors. As a consequence, the sensors battery may drain quickly and the multi-hop network connection may be lost  [22]. By utilizing the UAVs as mobile information collectors, sensors can directly send monitored information to UAVs. However, as UAVs have limited on-board battery, it is important to reduce the completion time needed for the information collection mission. In this paper, we aim to minimize the maximum completion time among all UAVs by optimizing the trajectories of UAVs to complete the information collection mission as soon as possible. We assume the UAVs fly at a fixed altitude. The UAV collects information from sensors via time-division multiple access (TDMA) that means the UAV only communicates with at most one sensor at each time. Meanwhile, we consider a scenario where ground users are mainly composed of sensors. The extension to the three-dimensional (3D) trajectory planning and other multiple access schemes will be left for our future work. For convenience, Table 1 provides major notations used in this paper.

The UAV and sensor sets are denoted as $U\overset{\Delta }{=}\{u_{1},...,u_{k}\}$ and $P\overset{\Delta }{\;=}\{p_{1},....,p_{n}\}$, respectively. Considering a 3D Cartesian coordinate system, sensor $p_{j}$ is fixed at $c_{j}=\left(x_{j},y_{j},0\right)$. Denote the trajectory of UAV $u_{i}$ projected on the ground as $l_{i}\left(t\right)={\left[x_{i}\left(t\right),y_{i}\left(t\right)\right]}^{T}\in$$R^{2\times 1}$, where 0≤t≤T. The trajectory of each UAV should satisfy the following constraint

(1) $$l_{i}\left(0\right)=l_{i}\left(T\right),\forall i,$$

which means that the UAVs need to fly back to their starting location by the end of monitoring mission. Furthermore, the trajectory of each UAV is subject to the velocity constraints, which can be given by

(2) $$∥\overset{\cdot }{l_{i}}\left(t\right)∥\leq v_{\max},\forall i,t\in \left[0,T\right],$$

where $v_{\max}$ denotes the maximum velocity of UAVs and $\overset{\cdot }{l_{i}}\left(t\right)$ is the time derivative of $l_{i}\left(t\right)$. As multi-UAV fly at the constant altitude, the trajectory of each UAV is expected to satisfy the collision avoidance constraint  [36]

(3) $$∥l_{i}\left(t\right)-l_{i^{\prime }}\left(t\right)∥\geq d_{\min},\forall i\neq i^{\prime },t\in \left[0,T\right],$$

where $d_{\min}$ is the minimum distance between the UAVs for avoiding collision. In addition, the time-varying distance between UAV $u_{i}$ and sensor $p_{j}$ can be expressed as

(4) $$d_{i,j}\left(t\right)=\sqrt{H^{2}+{\left(x_{i}\left(t\right)-x_{j}\right)}^{2}+{\left(y_{i}\left(t\right)-y_{j}\right)}^{2}}.$$

Due to scattering and ground reflection, the communication channels between UAVs and sensors include both Line-of-Sight (LoS) and Non-Line-of Sight (NLoS) links  [16]. The UAV–ground link channel is characterized by the presence of strong LoS path. The Rician channel model is an appropriate choice, as it can effectively reflect the combination of scattering and LoS that exists in the links between the UAVs and sensors  [13]. In this paper, we consider the more practically Rician fading channels between the UAVs and sensors  [13,16,37,38,39]. The instantaneous channel gains between the UAV $u_{i}$ and sensor $p_{j}$ can be expressed as  [16,37]

(5) $$h_{i,j}\left(t\right)=\sqrt{\beta_{i,j}\left(t\right)}g_{i,j},$$

where $g_{i,j}$ is the small-scale fading coefficient. $\beta_{i,j}\left(t\right)$ is the average channel power gain, which can be expressed by  [16,37]

(6) $$\beta_{i,j}\left(t\right)=\beta_{0}d_{i,j}^{-\alpha }\left(t\right)=\frac{\beta_{0}}{{\left[H^{2}+{\left(x_{i}\left(t\right)-x_{j}\right)}^{2}+\left(y_{i}\left(t\right)-y_{j}\right)\right]}^{\alpha /2}},$$

where α is the path loss exponent and $\beta_{0}$ denotes the average channel power gain at the reference distance $d_{0}=1m$. $g_{i,j}$ is a random variable that corresponds to the effects of the small-scale fading such that $E\left[{\left|g_{i,j}\right|}^{2}\right]=1$, which can be given by  [16,37]

(7) $$g_{i,j}=\sqrt{\frac{K_{R}}{K_{R}+1}}g+\sqrt{\frac{1}{K_{R}+1}}\tilde{g},$$

where $K_{R}$ is the Rician factors of the channel and *g* denotes the deterministic LoS channel component with $\left|g\right|=1$. $\tilde{g}$ represents the random scattered component which is a zero-mean unit-variance circularly symmetric complex Gaussian (CSCG) random variable. For Rician fading, the cdf of ${\left|g_{i,j}\right|}^{2}$ is explicitly represented as  [37,38]

(8) $$F_{{\left|g_{i,j}\right|}^{2}}\left(x\right)=1-Q_{1}\left(\sqrt{2K_{R}},\sqrt{2(K_{R}+1)x}\right),$$

where $Q_{1}\left(a,b\right)$ is the standard Marcum-Q function. We assume that *p* is the transmission power of sensors. The instantaneous channel capacity is expressed as  [36]

(9) $$\begin{array}{c} R_{i,j}\left(t\right)=B\log_{2}\left(1+\frac{p{\left|h_{i,j}\left(t\right)\right|}^{2}}{\sum_{m=1,m\neq j}^{n}p{\left|h_{i,j}\left(t\right)\right|}^{2}x_{m}\left(t\right)+\sigma^{2}}\right), \end{array}$$

where *B* is the channel bandwidth and $\sigma^{2}$ denotes the white Gaussian noise power at the receiver. The term $\sum_{m=1,m\neq j}^{n}p{\left|h_{i,m}\left(t\right)\right|}^{2}x_{m}\left(t\right)$ in Equation (9) represents the co-channel interference caused by the transmissions of other sensors in time *t*. We denote a binary variable $x_{m}\left(t\right)$ to indicate whether sensor $p_{m}$ transmits information to UAVs at time *t*, which can be given by

(10) $$x_{m}\left(t\right)=\left\{\begin{array}{c} 1\;p_{j}\;transmits\;information\;to\;UAVs\\ 0\;otherwise \end{array}\right..$$

The maximum communication radius projected on the ground is expressed by *r*, which depends on the maximum communication distance $d_{U-S}^{\max}$ and UAV altitude *H*, which is determined as  [15,40]

(11) $$r=\sqrt{d{{}_{U-S}^{\max}}^{2}-H^{2}},$$

We assume that the UAVs fly at a determined altitude *H* when they collect information from the sensors. Therefore, *r* mainly depends on the maximum communication distance. To successfully collect information from sensors, the received power of UAV must be higher than or equal to the minimum decodable power $p_{U,\min}$ [40]. The maximum communication distance between the sensor and the UAV can be calculated as

(12) $$d_{U-S}^{\max}=\sqrt{\frac{G_{S}^{t}\cdot G_{U}^{r}\cdot \lambda^{2}\cdot p}{{\left(4\pi \right)}^{2}\cdot p_{U,\min}}},$$

where $G_{S}^{t}$ is the transmit antenna gain of the sensor, $G_{U}^{r}$ is the receive antenna gain of the UAV. λ represents the wavelength of the signal transmitted by a sensor which is calculated by the frequency of signal. Based on the maximum communication radius *r*, the communication condition can be expressed as

(13) $$∥l_{i}\left(t\right)-{c_{j}}^{\prime }∥\leq r,$$

where ${c_{j}}^{\prime }$ is the coordinate representation of sensor $p_{j}$ on a two-dimensional plane. It is worth noting that, if UAV $u_{i}$ needs to collect information from sensor $p_{j}$ at time *t*, in order to ensure reliable transmission, the distance between them must satisfy Equation (13).

We define a binary variable $f_{i,j}\left(t\right)$, and it indicates whether sensor $p_{j}$ is served by UAV $u_{i}$ at time *t*

(14) $$f_{i,j}\left(t\right)=\left\{\begin{array}{c} 1\;u_{i}\;collect\;information\;from\;p_{j}\\ 0\;otherwise \end{array}\right..$$

The binary variables specify not only the communication scheduling across the different time, but also the association between UAVs and sensors. In this paper, the UAV collects information from sensors via TDMA that means the UAV only communicates with at most one sensor at each time. Meanwhile, each sensor is served by only one UAV at a time instance, but can be served by different UAVs over different time slots. These constraints can be expressed as

(15) $$\sum_{j=1}^{n}f_{i,j}\left(t\right)\leq 1,\forall i,j,$$

(16) $$\sum_{i=1}^{k}f_{i,j}\left(t\right)\leq 1,\forall i,j,$$

Furthermore, the total amount of data ${\bar{R}}_{j}$ transmitted from sensor $p_{j}$ to UAV $u_{i}$ is a function of UAV trajectory $l_{i}\left(t\right)$, which is expressed as

(17) $${\bar{R}}_{j}\left(l_{i}\left(t\right)\right)=\int_{0}^{T}B\log_{2}\left(1+\frac{p{\left|h_{i,j}\left(t\right)\right|}^{2}}{\sum_{m=1,m\neq j}^{n}p{\left|h_{i,j}\left(t\right)\right|}^{2}x_{m}\left(t\right)+\sigma^{2}}\right)f_{i,j}\left(t\right)dt.$$

### 3.2. Problem Formulation

The throughput requirement corresponding to sensor $p_{j}$ is assumed to be $C_{j}$ bits. By optimizing the trajectories of UAVs, our objective is to minimize mission completion time, while ensuring that the information of sensors is collected. The mission completion time refers to the time taken by all UAVs to complete respective tasks. The completion time of a UAV is mainly divided into two parts, flying time and hovering time. Therefore, the completion time of a single UAV can be represented as

(18) $$T_{i}=T_{F}^{i}+T_{H}^{i},$$

where $T_{F}^{i}$ represents the flying time and $T_{H}^{i}$ represents the hovering time.

We denote $F=\{f_{i,j}\left(t\right),\forall i,j,t\}$ and $L=\{l_{i}\left(t\right),\forall i,t\}$. Assuming the sensors’ locations are given, we aim to minimize the mission completion time by jointly optimizing the UAVs’ trajectories (i.e., L), communication scheduling and association (i.e., F). Define $T\left(F,L\right)=\max_{1\leq i\leq k}T_{i}$ as the mission completion time which is a function of F and L. The optimization problem can be formulated as

(19) $$\begin{array}{c} \min_{F,L,\left\{T_{H}\right\},\left\{T_{F}\right\}}\;\;\;T \end{array}$$

(20) $$\begin{array}{cc} s.t.\; & l_{i}\left(0\right)=l_{i}\left(T\right),\forall i, \end{array}$$

(21) $$\begin{array}{cc} \;\; & {\bar{R}}_{j}\geq C_{j},\forall j, \end{array}$$

(22) $$\begin{array}{cc} \;\; & \sum_{i=1}^{k}f_{i,j}\left(t\right)\leq 1,\forall i,j,t\in \left[0,T\right], \end{array}$$

(23) $$\begin{array}{cc} \;\; & \sum_{j=1}^{n}f_{i,j}\left(t\right)\leq 1,\forall i,j,t\in \left[0,T\right], \end{array}$$

(24) $$\begin{array}{cc} \;\; & ∥\overset{\cdot }{l_{i}}\left(t\right)∥\leq v_{\max},\forall i,t\in \left[0,T\right], \end{array}$$

(25) $$\begin{array}{cc} \;\; & ∥l_{i}\left(t\right)-l_{i^{\prime }}\left(t\right)∥\geq d_{\min},\forall i\neq i^{\prime },t\in \left[0,T\right], \end{array}$$

(26) $$\begin{array}{cc} \;\; & 0\leq ∥l_{i}\left(t\right)-{c_{j}}^{\prime }∥f_{i,j}\left(t\right)\leq r,\forall i,j,t\in \left[0,T\right]. \end{array}$$

This problem is challenging to be solved due to three main reasons. First, the problem needs to optimize continuous functions F and L, which essentially involve an infinite number of optimization variables that are closely coupled with each other. Second, since the optimization variables F for UAV–sensor association and communication scheduling are binary, the formulated problem is a non-convex problem. Third, it is difficult to optimize trajectories, hovering and flying communication scheduling at the same time. To solve this problem, we propose an improved fly-hover-fly trajectory planning algorithm (FLY), in which the UAVs successively hover at a finite number of hovering points each for an optimized hovering duration. Different from other traditional fly-hover-fly trajectory designs  [25,26,27,28,29,30], UAVs can collect information from sensors not only in hovering, but also in flying. First, we propose the FHF algorithm to optimize the hovering points and obtain the trajectories in Section 4. Then, under the obtained trajectories, we further consider the UAVs can also collect information when flying and optimize the time allocations in Section 5.

## 4. FHF Trajectory Planning Algorithm

**Figure 2:**
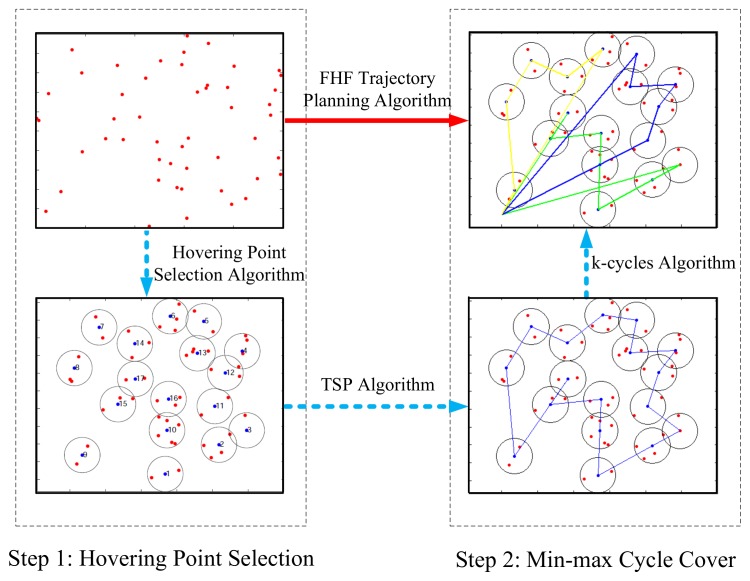
FHF trajectory planning algorithm.

As shown in Figure 2, we propose FHF algorithm which includes two steps. First, we propose a hovering point selection algorithm to select appropriate hovering points with considering the information collection quality. Red dots represent sensors and blue dots represent optimized hovering points. The number of hovering points is less than that of ground sensors. Not stopping above each sensor node, we select appropriate hovering points where the UAVs can sequentially collect the information from multiple sensors. By hovering at different points, which are close to different subsets of sensors, the UAVs can obtain better wireless communication links and decrease the flying time compared with the policy of hovering above each sensor. Moreover, the policy of hovering above each sensor was used as a comparison algorithm in our simulations.

Second, we use min-max cycle cover algorithm to assign these hovering points and get the trajectory of each UAV. Based on the hovering points optimized in the first step, we use Traveling Salesman Problem (TSP) algorithm to calculate a cycle covering all hovering points. Then, we use a novel k-cycles algorithm for getting the trajectory of each UAV, which can minimize the maximum time of the cycles including flying time and hovering time.

### 4.1. Hovering Point Selection

For a given SNR threshold, it is unnecessary for UAVs to fly over all sensors in general. We model hovering point selection problem as a BS coverage problem  [15]. Specifically, given sensors’ locations and UAV’s communication radius, the hovering point selection problem’s objective is to obtain a minimum number of hovering points and respective locations, while ensuring that all sensors are covered by at least one hovering point. As shown in Algorithm 1, we use the min-max location algorithm as sub-algorithm to solve this problem. Meanwhile, major notations used in Algorithm 1 are provided in Table 2.

**Algorithm 1** Hovering point selection algorithm.
**Input**: *r*, sensor set *P* with known locations ${\left\{c_{j}\right\}}_{p_{j}\in P}$.**Output**: Hovering point set *V*, with optimized locations ${\left\{h_{m}\right\}}_{v_{m}\in V}$.1:Initialize X←∅, Y←P, V←∅, m←1;2:
**while** $\left|X\right|<n$ **do**
3: Calculate boundary sensor set $Y_{bo}\subseteq Y$, update inner sensor set $Y_{in}\leftarrow Y\setminus Y_{bo}$;4: Randomly select a sensor $b_{0}\in Y_{bo}$. Denote $\rho \leftarrow \{b\left|∥b_{0}-b∥\right.\leq 2r,b\in Y_{bo}\}$;5: Use the min-max location algorithm to find hovering point’s location $h_{m}$ to cover ρ. Denote the maximum distance is *d*;6: **while** d>r **do**7:  $\rho \leftarrow \rho \setminus \arg\max_{b\in \rho }∥b-h_{m}∥$. Repeat step 5;8: **end while**
9: $\rho \leftarrow \rho \cup \{b\left|∥b-h_{m}∥\right.\leq r,b\in Y_{in}\}$;10: $\theta \leftarrow \{b\left|r<∥b-h_{m}∥\right.\leq 2r,b\in Y_{in}\}$;11: **while** θ≠∅ **do**12:  Find a sensor $b^{\prime }\in \theta$ which has the shortest distance to $h_{m}$;13:  Remove (add) $b^{\prime }$ from (to) θ (ρ) if $b^{\prime }$ is covered by changing $h_{m}$ via using min-max location algorithm. Stop otherwise;14: **end while**
15: X←X∪ρ;16: Y←Y∖ρ;17: $V\leftarrow V\cup \{v_{m}\}$, m←m+1.18:
**end while**



Now, we describe the hovering point selection algorithm in detail. First, we define *Y* as the set of uncovered sensors, which is initialized to be equal to *P*; *X* as the set of covered sensor, which is initialized to an empty set; and *V* as the set of hovering points, which is initialized to empty set (Line 1). We utilize the convex hull to define boundary sensors, whereas other boundary sensors definitions can also be used to produce similar results. In this algorithm, to reduce the occurrence of outlier sensors, which require one hovering point to cover it, we give higher priority to the boundary sensors so that boundary sensors are guaranteed to be covered firstly. The boundary sensors exist in $Y_{bo}$ and the inner sensors exist in $Y_{in}$ (Line 3). We randomly choose a boundary sensor $b_{0}$ that exists in $Y_{bo}$. The sensors that are less than 2r away from $b_{0}$ are put in ρ (Line 4).

At this time, it can be converted into the min-max location problem to find the position of the hovering point. In operations research of facilities location type, the min-max location problem is a classical combinatorial optimization problem. The problem can be described as follows: given a function to calculate the cost between demand points and a facility, a space of feasible locations of a facility, and a set of *n* demand points, find the facility’s location which minimizes the maximum facility-demand point cost [41,42]. The simple special case when the demand points and feasible locations are in the plane with Euclidean distance as cost, it is also known as the smallest circle problem [43]. Our goal is to minimize the maximum sensor-hovering point distance. Therefore, we use the min-max location algorithm to find hovering point’s location $h_{m}$ to cover all sensors in ρ [44] (Line 5). This algorithm minimizes the maximum distance from sensors to $h_{m}$. The farthest distance is defined as *d*.

**Figure 3:**
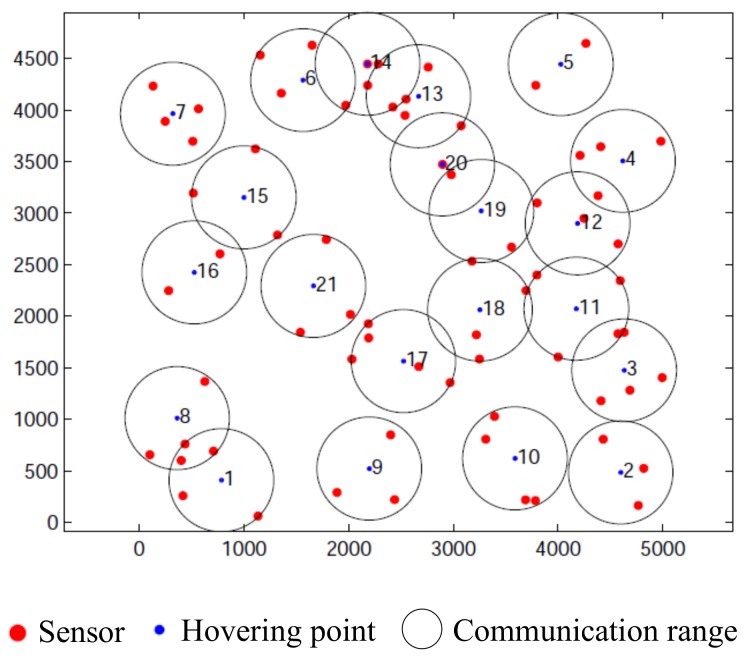
Hovering points.

If the value of *d* exceeds *r*, ρ needs to be rebuilt until the farthest distance is less than *r*. In each iteration, the sensor which is the farthest away from $h_{m}$ is deleted (Lines 6–8). Update ρ by adding sensors which satisfy the distance conditions (Line 9). This check reduces the execution times of the min-max location algorithm in the next step. The sensors in $Y_{in}$ whose distance to $h_{m}$ are in the range of (r,2r) are put in θ (Line 10). We find a sensor $b^{\prime }\in \theta$ which is the closest to $h_{m}$. Remove (add) $b^{\prime }$ from (to) θ (ρ) if $b^{\prime }$ is covered by changing $h_{m}$ via using min-max location algorithm. When θ is empty or new $h_{m}$ is not found, the *m*th hovering point’s location $h_{m}$ is determined (Line 11–14). *X*, *Y* and *V* are updated based on the results we got (Line 15–17). When all sensors are covered, the algorithm will stop. In Figure 3, we show an example of Algorithm 1. There are seventy sensors in a square area of 5 km × 5 km and *r* equals 500 m. By utilizing Algorithm 1, we obtain twenty hovering points and respective locations.

For hovering point selection algorithm, the convex hull algorithm of *n* points to find boundary sensors has complexity O(nlogn) [15,45]. In addition, each hovering point executes the min-max location algorithm for up to O(n) times. Meanwhile, the complexity of the min-max location algorithm is $O(n^{2})$ [44]. Since the number of hovering points is at most O(n), the overall complexity of the hovering point selection algorithm is upper-bounded by $O(n\left[n\log n+n^{3}\right])$. To sum up, the complexity of the hovering point selection algorithm is upper-bounded by $O(n^{4})$.

### 4.2. Min-Max Cycle Cover

The multi-UAV system is modeled as a complete undirected graph G=(V,E), where vertices in *V* represent hovering points for UAV and edges in *E* represent flying paths of the UAV. For each edge $e(v_{m},v_{m+1})\in E$, $v_{m},v_{m+1}\in V$, an edge weight $w(v_{m},v_{m+1})$ is given to represent the flying time. For each vertex $v_{m}$, a vertex weight $h(v_{m})$ is given to represent the hovering time to collect information. Since we use a multi-UAV system to collect information, the completion time of the entire mission is the time of tasks completed by all UAVs. In other words, it is the maximum completion time of UAVs. The minimum mission completion time problem aims to cover hovering points with multi-UAV such that the maximum weight of the cycles including flying time and hovering time is minimized. To tackle this problem, we formulate it as a min-max cycle cover problem. The min-max cycle cover problem is an extension of the classical TSP. Since the TSP is NP-hard when k=1, the min-max cycle cover problem is also NP-hard for any k≥1 [46].

The heuristic algorithm we propose includes two steps to plan the trajectory of each UAV. Firstly, the ant colony algorithm is used for obtaining a cycle *C* covering all hovering points. As a global searching algorithm, the ant colony algorithm can solve TSP problems [47]. Then, we use a novel *k*-cycles algorithm for getting the trajectory of each UAV [48]. This algorithm mainly decomposes cycle *C* into *k* segments.

**Figure 4:**
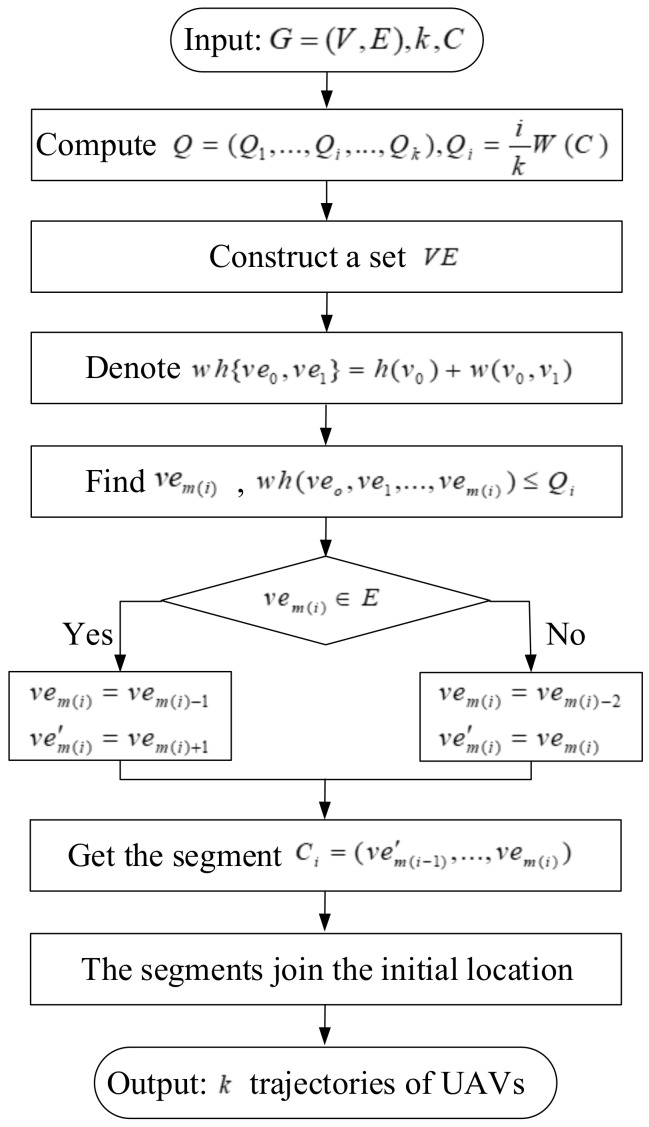
*k*-cycles algorithm.

The *k*-cycles algorithm for obtaining *k* trajectories of UAVs is shown as Figure 4. This algorithm takes the number of UAVs, cycle *C* and the complete undirected graph G=(V,E) as input. In *k*-cycles algorithm, to decompose cycle *C* into *k* trajectories of UAVs, we first compute the bound vector $Q=(Q_{1},...,Q_{i},...,Q_{k})$. $Q_{i}$ can be calculated on the basis of the total weight of cycle *C*, $Q_{i}=\frac{i}{k}W\left(C\right)$, for all 1≤i≤k, where W(C)=h(C)+w(C). h(C) and w(C) represent the weight of all vertices and the weight of all edges in cycle *C*, respectively. Second, along the cycle, we build a mixed set of edges and vertices containing $2\left|V\right|+1$ elements, denoting it as $VE=(ve_{0}=v_{0},ve_{1}=e\left(v_{0},v_{1}\right),ve_{2}=v_{1},ve_{3}=e\left(v_{1},v_{2}\right),...,ve_{2\left|V\right|+1}=v_{0})$. $wh(ve_{0},...,ve_{m})$ represents the sum weight of vertices and edges. Compared with [49], the weight of vertices, which represents the hovering time, is calculated one time in *k*-cycles algorithm. By constructing a mixed set of vertices and edges, the weight of vertices can be taken into account in the cycle splitting. Next, from ${ve}_{0}$, we aim to find an element ${{ve}_{m}}_{\left(i\right)}$ along cycle *C* such that the weight of segment $wh(ve_{o},ve_{1},...,ve_{m(i)})$ satisfies $wh\left(ve_{o},ve_{1},...,ve_{m(i)}\right)\leq Q_{i}$, for each *i*, 1≤i≤k. There are two possibilities for the demarcation point ${{ve}_{m}}_{\left(i\right)}$.

- If $ve_{m(i)}$ is a path, we would delete this path when we decompose the cycle. Then, we define $ve_{m(i)-1}$ as the ending point of this segment, $ve_{m(i)+1}$ as the starting point of the next segment.
- If $ve_{m(i)}$ is a hovering point, we would define it as the starting point of the next segment (i.e., $v{e^{\prime }}_{m(i)}=ve_{m(i)}$). Then, we take $ve_{m(i)-2}$ as the end of this segment.

Then, we obtain *k* segments of cycle *C* (i.e., $\left\{C_{1},...,C_{i},...,C_{k}\right\}$). Finally, the segments add the starting location to build the closed trajectory for each UAV.

The complexity of the ant colony algorithm is related to the number of ants, the number of hovering points and the number of iterations. The number of hovering points cannot exceed *n*. Thus, the complexity of the ant colony algorithm is upper-bounded by $O(iter\cdot A\cdot n^{2})$, where iter is the number of iterations and *A* is the number of ants. Furthermore, the complexity of *k*-cycles algorithm depends on the number of sensors. Since *k*-cycles algorithm is a polynomial-time algorithm, its complexity is O(n). In addition, the complexity of the hovering point selection algorithm is upper-bounded by $O(n^{4})$. To sum up, the complexity of the hover-collection UAV trajectory planning algorithm is upper-bounded by $O(n^{2}\left[n^{2}+iter\cdot A\right])$.

## 5. Time Allocation

With the obtained UAVs trajectories, we further consider the UAVs can also collect information when flying. We propose an effective method to optimize UAVs fly-collection and hovering time allocations.

We define ${\tilde{T}}_{H,m}\geq 0$ as the new allocated time for the UAVs to collect information when hovering at location $v_{m}$. Since UAVs can collect information when flying, we can obtain a constraint ${\tilde{T}}_{H,m}\leq T_{H,m},\forall m$. The time allocation problem is challenging to be solved due to three main reasons. First, the time allocation problem needs to optimize continuous functions F, which essentially involves an infinite number of optimization variables. Second, the mission completion time is unknown. Therefore, the time allocation problem cannot be solved by widely time discretization method. Third, the UAV–sensor association and communication scheduling are binary, thus the formulated problem is a non-convex problem. To tackle this problem, we use a new discretization method, called path discretization [50,51].

With the UAVs’ trajectories UT and hovering points *V* given, UAV’s trajectory $UT_{i}$ can be discretized into $Z^{i}$ line segments, which are represented by the $Z^{i}$ sampling points $\left\{q_{z^{i}}\right\}$. The length of the $z^{i}$th line segment can be expressed as $\mu_{z^{i}}=∥q_{z^{i}+1}-q_{z^{i}}∥$. Note that $Z^{i}$ needs to be chosen to be sufficiently large so that $\mu_{z^{i}}\leq \mu_{{}_{\max}}^{i}$. $\mu_{{}_{\max}}^{i}$ is an appropriately chosen value so that the distance between each sensor and UAV $u_{i}$ is approximately unchanged under each line segment. Furthermore, we assume the time for flying in the $z^{i}$th line segment is $\pi_{z^{i}}$. Meanwhile, $\lambda_{z^{i}}$ is defined as the allocated information collection time when $u_{i}$ flies on the $z^{i}$th line segment, where $\lambda_{z^{i}}\leq \pi_{z^{i}}$. When information collection occurs in the mobile state, the UAV sends an activation signal to a sensor which satisfies SNR conditions. Then, the UAV collects information from the activated sensor.

The formulated problem reduces to optimizing the communication scheduling F, the hovering time $\left\{{\tilde{T}}_{H}\right\}$ and fly-collection time $\left\{\lambda_{z^{i}}\right\}$. Therefore, we can obtain

(27) $$\begin{array}{c} \min_{F,\left\{{\tilde{T}}_{H}\right\},\left\{\lambda_{z^{i}}\right\}}\;\;\;T \end{array}$$

(28) $$\begin{array}{cc} s.t.\; & {\bar{R}}_{j}\geq C_{j},\forall j, \end{array}$$

(29) $$\begin{array}{cc} \;\; & \sum_{i=1}^{k}f_{i,j}\left(t\right)\leq 1,\forall i,j,t\in \left[0,T\right], \end{array}$$

(30) $$\begin{array}{cc} \;\; & \sum_{j=1}^{n}f_{i,j}\left(t\right)\leq 1,\forall i,j,t\in \left[0,T\right], \end{array}$$

(31) $$\begin{array}{cc} \;\; & 0\leq ∥l_{i}\left(t\right)-{c_{j}}^{\prime }∥f_{i,j}\left(t\right)\leq r,\forall i,j,t\in \left[0,T\right], \end{array}$$

(32) $$\begin{array}{cc} \;\; & {\tilde{T}}_{H,m}\leq T_{H,m},\forall m, \end{array}$$

(33) $$\begin{array}{cc} \;\; & \lambda_{z^{i}}\leq \pi_{z^{i}},\forall z,i. \end{array}$$

With the given trajectories, since the velocity of UAVs remains unchanged, the new hovering time cannot exceed the initial value. The new formulated problem is an integer programming problem, which can use the existing software toolbox such as CVX to be efficiently solved. Therefore, under the given trajectories, we can get fly-collection and hovering time allocations. Furthermore, joint optimization of multi-UAV trajectories, communication scheduling and time allocation will be left for our future works.

## 6. Simulation Results

In this section, we present the simulation results to validate the proposed FLY algorithm, which mainly includes the FHF algorithm and time allocations.

### 6.1. Simulation Setup

We assumed that the size of the monitoring area was 5 km × 5 km. The total bandwidth was 1 MHz, and the average channel power gain at 1 m was −50 dBm [52]. The maximum velocity of a UAV was set to 50 m/s. Furthermore, the minimum distance between UAVs was set to 100 m [36] and UAVs flew at a constant altitude of 50 m. We assumed that the transmission power equaled 10 dBm [16]. The white Gaussian noise power was equal to −110 dBm [16]. Following the authors of [15,40], the communication radius was 500 m, which was calculated by the altitude and maximum communication radius. As stated in [36], the Rician channel model can be utilized for estimating the link performance for UAV-to-ground links. Simulations were conducted using the parameters specified in Table 3.

### 6.2. Baseline Setup

To demonstrate the performance of the proposed information-collection UAV trajectory planning algorithm (FLY), we compared and implemented the following six benchmark schemes:

- FHF algorithm: It is the subalgorithm of the FLY algorithm. This algorithm only considers the UAVs collecting information when they are hovering.
- Hovering above each sensor (SHP): The feasible trajectory of the UAV needs to ensure the throughput requirement of each sensor is satisfied. One straightforward approach is to let the UAVs sequentially visit all sensors. First, SHP algorithm calculates a cycle which covers all sensors using TSP algorithm. Then, it uses *k*-cycles algorithm to obtain the trajectories of UAVs.
- Sensor balanced algorithm (PB): This algorithm first calculates a cycle by using TSP algorithm. Then, it decomposes the cycle into *k* trajectories that contain the same number of sensors.
- KTSP-based algorithm (KTSP): This algorithm designs the trajectories of multi-UAV by using K-Traveling Salesman Problem (KTSP) algorithm [53].
- Kmeans-based algorithm (KMEAN): This algorithm uses kmeans algorithm to obtain hovering points. Then, it uses the min-max cycle cover algorithm to obtain the trajectories of UAVs [54].
- Optimal scheme (OPT): To evaluate how the proposed algorithms approach the optimal performance, we use brute-force searching method to obtain the optimal scheme that minimizes the mission completion time.

### 6.3. Different Number of Sensors

**Figure 5:**
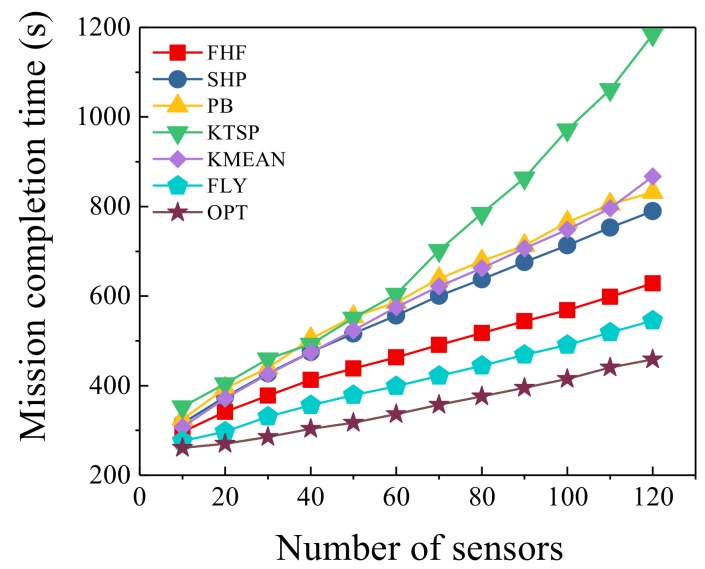
Different number of sensors.

In this simulation, with different number of sensors, the number of UAVs was fixed at three and the seven algorithms were compared. As the number of sensors changes, the trend of mission completion time is shown in Figure 5. It was observed that the algorithms we proposed significantly outperformed four other algorithms. FHF and FLY algorithms were the closest to the optimal solution compared with four other algorithms. FLY algorithm achieved almost the same performance with the optimal scheme when n<20, and the mission completion time difference was less than 6%. When the number of sensors was greater than 20, FLY algorithm was also closer to the optimal scheme than five other algorithms. For example, when n=120, FLY algorithm was 119% of the optimal solution, which was the biggest gap between FLY algorithm and the optimal scheme in this simulation. It was also observed that the completion time of mission conducted only in static case cOULD not be smaller than in both static and mobile cases. FLY algorithm was reduced by 7.0–14.1% compared with FHF algorithm. That was expected; since UAVs utilized the flying phase to collect information, the hovering time was reduced. Meanwhile, we found that our subalgorithm (FHF) performed better than the other compared algorithms. FHF algorithm was reduced by 3.5–46.9% compared with four other algorithms. When the number of sensors was 10, FHF algorithm was reduced by 3.5% compared with KMEAN algorithm. When the number of sensors was 120, FHF algorithm was reduced by 46.9% compared with KTSP algorithm. By hovering at a point where it could communicate with multiple sensors, the UAV could decrease the flying time compared with SHP algorithm, PB algorithm and KTSP algorithm. In particular, when the number of sensors increased, the UAV could communicate with more sensors at one hovering point. Therefore, our algorithms performed better when the number of sensors was large. Moreover, SHP algorithm and PB algorithm use the same TSP algorithm, thus the performance was relatively close. Meanwhile, KMEAN algorithm selected hovering points without considering the information collection quality of UAVs. There was retransmission in the actual transmission, which increased the information collection time and KMEAN algorithm did not reach the effect of selecting the hovering points. Therefore, KMEAN algorithm was close to SHP algorithm and PB algorithm.

### 6.4. Different Number of UAVs

**Figure 6:**
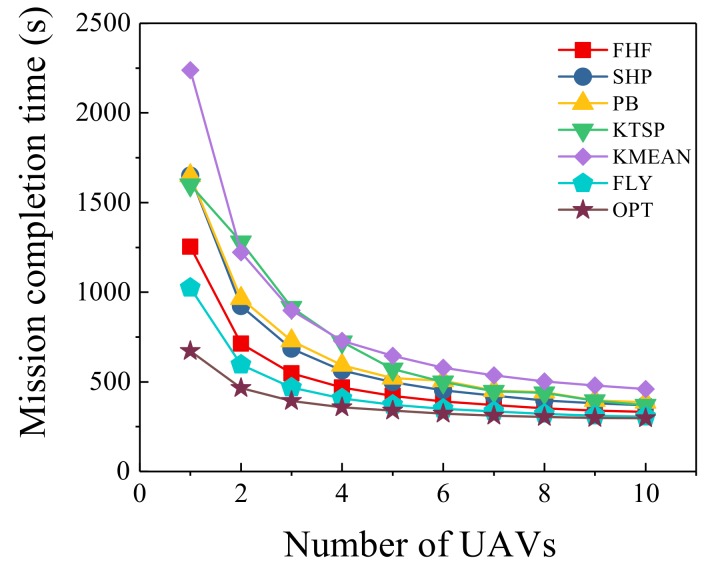
Different number of UAVs.

Under different number of UAVs, the number of sensors was set to be 100 and we compared the seven algorithms. As the number of UAVs changes, the trend of mission completion time is shown in Figure 6. Obviously, increasing the number of UAVs could improve the speed of monitoring, and the mission could be completed in less time. However, increasing the number of UAVs also brought some overhead and economic pressure. As shown in Figure 6, from one UAV to four UAVs, the mission completion time dropped significantly. When the number of UAVs exceeded five, the changes in mission completion time tended to be flat. It was obvious that more UAVs could share the monitoring mission to reduce mission completion time. However, some sensors were far from the initial location, and the UAV might take too much time going back and forth. Therefore, the maximum completion time of UAVs had a lower bound, and it was impossible to keep falling as the number of UAVs increased. Compared with four other algorithms, our algorithms were closer to optimal scheme. As shown in Figure 6, the gap between our algorithms and the optimal solution decreased as the number of UAVs increased. When k=1, FHF algorithm was 86.7% higher than the optimal scheme and FLY algorithm was 52.4% higher than it. When k=10, FHF algorithm was 12% higher than the optimal scheme and FLY algorithm was 3.1% higher than it. As the number of UAVs changed, FLY algorithm was reduced by 8.0–18.1% compared with FHF algorithm. Such results demonstrate the flying and hovering information collection solution is better than hovering information collection solution.

### 6.5. Different Communication Radius

**Figure 7:**
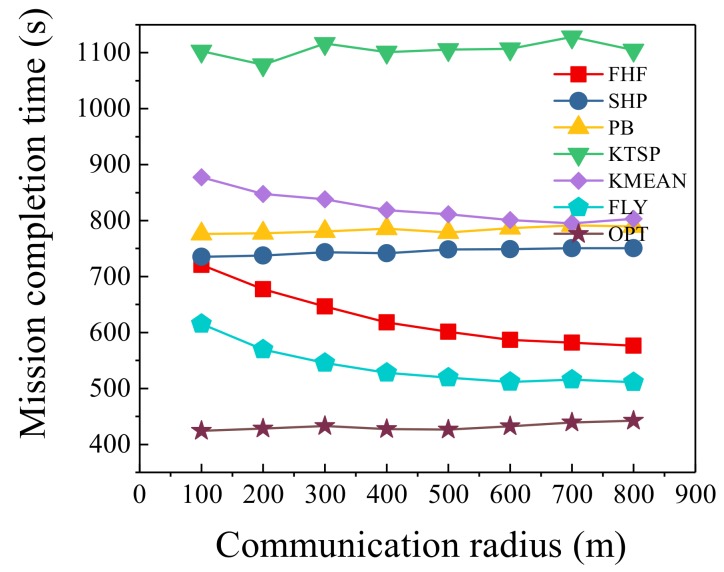
Different communication radius.

Under different communication radius, we set the number of UAVs at 3 and the number of sensors at 120, and compared the seven algorithms. Figure 7 shows the trend of mission completion time as the communication radius changes. Under different communication radius, our algorithms were closer to the optimal scheme than four other algorithms. As the communication radius increased, our algorithms had obvious advantages. When the communication radius was 100 m, FLY algorithm was 145% of the optimal algorithm, and FHF algorithm was 169% of the optimal algorithm. However, when the communication radius was 800 m, FLY algorithm was 116% of the optimal algorithm, and FHF algorithm was 130% of the optimal algorithm. As shown in Figure 7, the mission completion time of our algorithms and KMEAN algorithm gradually decreased as the communication radius increased, and other algorithms’ change trends were relatively small. The reason is that the change in communication radius mainly affected the hovering point selection algorithm, which is an important difference between three algorithms and three other algorithms. Since we considered the information collection quality in hovering point selection algorithm, our algorithms had a higher performance improvement compared with KMEAN algorithm. Of course, in practical applications, the communication range of the UAV is related to the altitude and the communication equipment of the UAV.

### 6.6. Different Monitoring Area Size

**Figure 8:**
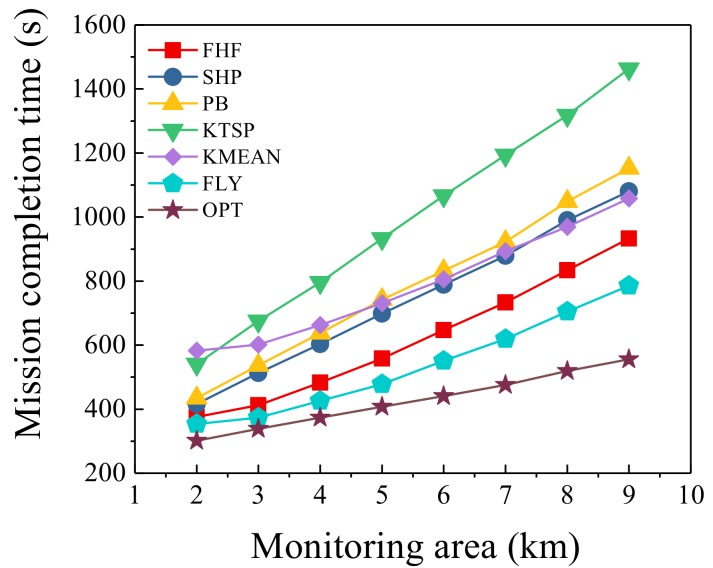
Different monitoring area size.

Under different monitoring area size, we set the number of UAVs at 3 and the number of sensors at 100 and compared the seven algorithms. Figure 8 shows the trend of mission completion time as the monitoring area size changes. It was observed that FHF and FLY algorithms were closer to the optimal solution than four other algorithms. The gap between FHF algorithm and the optimal solution increased as the monitoring area expanded, from 21.6% to 67.7%. The gap between FLY algorithm and the optimal solution increased as the monitoring area expanded, from 10.3% to 41.3%. As the monitoring area became larger, the advantage of FLY algorithm was more obvious. FLY algorithm was reduced by 6–15.6% compared with FHF algorithm. That was expected; since the larger area caused a long flying time, the UAVs could obtain more time to collect information when flying. Furthermore, FHF algorithm was reduced by 9.7–40.2% compared with four other algorithms. As the monitoring area size increased, the flying scope of the UAV became larger, and the flying time became longer, resulting in longer mission completion time. As shown in Figure 8, FLY algorithm performed the best under different monitoring area size. Since it fully considers the information collection quality and the positions of sensors, the algorithm can be applied to different monitoring area size. Based on the simulation results in the previous subsection, if the communication range of UAVs is appropriately changed, the mission completion time will be shorter.

## 7. Discussion

### 7.1. Trade-Offs in Communication and Trajectory Design

There are some new and interesting trade-offs among the energy, delay and throughput in UAV communication and trajectory design which are different from traditional terrestrial communication [55]. In this section, we mainly discuss the trade-offs of UAVs when they are used as mobile information collectors. First, there is a trade-off between delay and throughput in UAV-enabled wireless network. To maximize throughput, UAVs always fly sufficiently close to the sensors to improve the link capacity. Although this method improves the throughput, it also brings delay due to the movement of the UAV. Second, there is also an interesting trade-off between energy consumption and throughput in UAVs. To gain higher throughput, UAVs generally fly close to sensors which consume more energy to move. Third, the above two trade-offs naturally imply an energy-delay trade-off. To reduce the delay in UAV–sensor communication, UAVs need to move faster to sensors, which brings more energy consumption. In fact, by planning the trajectory of UAV, the energy, delay and throughput can be traded off among each other. Finally, there is a trade-off between sensors and UAVs [9]. The sensors have limited battery and lower power. To prolong the lifetime of sensors, UAVs can move close to sensors to collect their information with minimum transmit power. However, UAVs will consume more energy to move. In this paper, we mainly focus on minimizing mission completion time on the premise of achieving throughput requirements. In the future, we will consider these trade-offs in trajectory design.

### 7.2. Multiple Access Schemes

In this paper, we consider that the UAVs collect information from sensors via time-division multiple access (TDMA) that means only one sensor is scheduled for information collection at each time [36,37,39,55]. There are some works using other multiple access schemes [25,52]. For example, the authors considered a scenario that a UAV is dispatched as the mobile BS to provide service for ground users via orthogonal frequency division multiple access (OFDMA) [52]. Besides orthogonal multiple access schemes such as TDMA considered above, non-orthogonal multiple access schemes also are considered in UAV-enabled wireless networks. To further improve the capacity limits, Wu et al. found that non-orthogonal multiple access schemes based on superposition coding (SC) can be jointly designed with the UAV trajectory [25]. The authors considered a two-user broadcast channel. To achieve the capacity region, they proposed a practical fly-hover-fly trajectory with SC. However, they only considered two users. In the future, for the next-generation wireless networks with massive connectivities, we will study designing the efficient trajectory algorithm joint with other multiple access schemes.

### 7.3. The Case of a Large Number of Sensors

In practical application, there may be many sensors deployed in the monitoring area. The complexity of hovering point selection problem becomes extremely high when the number of points *n* is very large. Therefore, the application scope of the proposed algorithm will be limited. To expand the application scope of the algorithm, we propose an efficient method. When the number of sensors is large, the monitoring area can be divided into some subareas. In other words, ground sensors can be partitioned into *M* disjoint sets, $P_{1},P_{2},$…$,P_{M}$ with each set corresponding to the sensors in its subarea. Then, the hovering point selection algorithm can be implemented for sensors in each subarea. By dividing the monitoring area, the complexity of trajectory planning problem can be reduced, and the application scope of the proposed algorithm can be improved.

## 8. Conclusions

In this paper, we aim to minimize the mission completion time of multi-UAV by optimizing the trajectories of UAVs to complete the mission as soon as possible. We formulate this problem as a mixed-integer non-convex problem. It is difficult to optimize trajectories, hovering and flying communication scheduling at the same time. To tackle the formulated problem, we propose an improved fly-hover-fly trajectory planning algorithm, which includes two steps. First, we propose the FHF algorithm to optimize the hovering points and obtain the trajectories. Second, with the obtained UAVs trajectories, we further consider that the UAVs can also collect information when flying. We propose an effective method to optimize UAVs fly-collection and hovering time allocations. The simulation results show that the mission completion time of our algorithm is minimum compared with other algorithms.

Since we add some constraints in the model, there are some limitations in practical application. For example, we assume that the UAVs fly at a fixed altitude. In fact, UAV’s altitude will directly affect Rician factor and information transmission rate. It is also worthwhile to optimize UAV’s altitude. In the future, we will exploit the vertical trajectory of the UAV and present a new design framework of 3D UAV trajectory to further improve the performance of multi-UAV information collection system. In addition, for the next-generation wireless networks with massive connectivities, we will study designing the efficient trajectory algorithm joint with other multiple access schemes. In our future works, other practical constraints on UAV’s trajectory will be considered, such as the maximum acceleration, the maximum instantaneous output power of the engine and the maximum turning angle.

## Figures and Tables

**Table 1:** Major notations.

Notation	Definition
*U*	UAV set
*P*	Sensor set
$u_{i}$	UAV *i*
$p_{j}$	Sensor *j*
*k*	Number of UAVs, or number of trajectories
*n*	Number of sensors
$l_{i}\left(t\right)$	$u_{i}$ trajectory projected on the ground
$\overset{\cdot }{l_{i}}\left(t\right)$	Time derivative of $l_{i}\left(t\right)$
$c_{j}$	Position of $p_{j}$
$d_{i,j}\left(t\right)$	Distance between UAV *i* and sensor *j*
$v_{\max}$	Maximum velocity
$d_{\min}$	Minimum inter-UAV distance
h(t)	Instantaneous channel power gain
$R_{i,j}\left(t\right)$	Instantaneous channel capacity
${\bar{R}}_{j}$	Total amount of data of $p_{j}$
*B*	Bandwidth
*p*	Transmission power
$\sigma^{2}$	White Gaussian noise power
*H*	Altitude of UAV
$f_{i,j}\left(t\right)$	Binary variable
*r*	Communication radius
$T_{F}^{i}$	Flying time
$T_{H}^{i}$	Hovering time
$T_{i}$	Completion time of $u_{i}$
*T*	Mission Completion time

**Table 2:** Major notations in Algorithm 1.

Notation	Definition
*V*	Hovering point set
*X*	Covered sensor set
*Y*	Uncovered sensor set
$h_{m}$	Hovering point’s location
$Y_{bo}$	Boundary sensor set
$Y_{in}$	Inner sensor set
$b_{o}$	A boundary sensor
*d*	The farthest distance to $h_{m}$

**Table 3:** Simulation parameters.

Definition	Parameters	Values
Monitoring area size	*D*	5 km× 5 km
Altitude of UAV	*H*	50 m
Maximum velocity	$v_{\max}$	50 m/s
Minimum inter-UAV distance	$d_{\min}$	100 m
Path loss	α	2.6
Bandwidth	*B*	1 MHz
Transmission power	*p*	10 dBm
Unit channel power gain	$\beta_{0}$	−50 dBm
White Gaussian noise power	$\sigma^{2}$	−110 dBm
Rician factor	$K_{R}$	2
Minimum decodable power	$p_{U,\min}$	−84 dBm
Frequency of signal	$f_{\lambda }$	2.4 GHz
Transmit antenna gain	$G_{S}^{t}$	1
Receive antenna gain	$G_{U}^{r}$	1
